# Deep learning-based fusion of nuclear segmentation features for microsatellite instability and tumor mutational burden prediction in digestive tract cancers: a multicenter validation study

**DOI:** 10.1093/bib/bbaf580

**Published:** 2025-11-11

**Authors:** Yanping Zhang, Jiaying Han, Huang Chen, Fengyuan Hu, Yaping Huang, Geng Tian, Dingrong Zhong, Jialiang Yang

**Affiliations:** School of Mathematics and Physics, Hebei University of Engineering, 19 Taiji Road, Handan 056038, China; School of Mathematics and Physics, Hebei University of Engineering, 19 Taiji Road, Handan 056038, China; Department of Pathology, China-Japan Friendship Hospital, 2 East Yinghuayuan Street, Beijing 100029, China; School of Mathematics and Physics, Hebei University of Engineering, 19 Taiji Road, Handan 056038, China; School of Mathematics and Physics, Hebei University of Engineering, 19 Taiji Road, Handan 056038, China; Geneis Beijing Co., Ltd., 31 Xinbei Road, Beijing 100102, China; Department of Pathology, China-Japan Friendship Hospital, 2 East Yinghuayuan Street, Beijing 100029, China; Geneis Beijing Co., Ltd., 31 Xinbei Road, Beijing 100102, China; Academician Workstation, Changsha Medical University, 1501 Leifeng Road, Changsha 410219, China

**Keywords:** cell nucleus segmentation, deep learning, gastric cancer, colorectal cancer, microsatellite instability (MSI), tumor mutational burden (TMB)

## Abstract

Microsatellite instability (MSI) and tumor mutational burden (TMB) are crucial biomarkers in gastric (GC) and colorectal cancer (CRC), yet their conventional sequencing-based detection is costly and time-consuming. Since only ~20% of patients are MSI-high or TMB-high and likely to benefit from immunotherapy, expensive genomic testing is often unjustified. This study developed a deep learning framework to predict MSI and TMB status directly from routinely available Hematoxylin and Eosin (H&E)-stained whole-slide images, leveraging fused nuclear segmentation features to improve accuracy. Using samples from TCGA (350 GC and 376 CRC for MSI; 400 GC and 387 CRC for TMB), image features were extracted with CLAM and nuclear features with Hover-Net. These features were combined via Multimodal Compact Bilinear Pooling and utilized in six distinct deep learning models. By fusing the nucleus segmentation features, the model increased area under the receiver operating characteristic curve (AUC) by 1%–3% and recall by 5%–11% in five-fold cross-validation, significantly outperforming models that relied solely on image features. External validation on a CRC dataset from the China-Japan Friendship hospital further validated the model's robustness, achieving an AUC of 0.81 and a recall of 0.80 for MSI prediction. Additionally, notable differences in cellular composition were observed across cancer types and clinical groups, emphasizing the pivotal role of cellular features in cancer development. These findings highlight the advantages of integrating H&E-stained image features with nuclear segmentation data and advanced deep learning techniques to improve predictive accuracy and reduce the cost of MSI/TMB testing, potentially advancing personalized cancer treatment strategies.

## Introduction

Cancer is widely recognized as one of the leading causes of death worldwide. Colorectal cancer (CRC) and gastric cancer (GC) are common malignant tumors of the digestive system. Among them, CRC ranks third in terms of incidence (9.6%) and second in terms of mortality rate (9.3%) [1–3]. GC ranks fifth in both incidence (4.9%) and mortality rate (6.8%) [4]. Traditional cancer treatment often leads to insufficient or failed treatment due to the possibility of disrupting normal dividing cells and being limited by side effects [5]. In recent years, immunotherapy based on immunoassay inhibitors (ICIs) has shown significant progress in treating various cancers [6]. However, the tumor microenvironment (TME) and immune system status vary significantly among patients, leading to different responses to immunotherapy [6, 7]. Additionally, immunotherapy is usually expensive. Therefore, in order to maximize the effectiveness of immunotherapy and reduce healthcare costs, it is critical to identify suitable biomarkers that can accurately guide immunotherapy selection. These biomarkers can help doctors identify which patients are most likely to benefit from ICI treatment, enabling personalized treatment and improving the treatment accuracy and efficacy.

Microsatellite instability (MSI), which affects DNA replication and repair mechanisms, is an important prognostic biomarker for various cancers [8, 9]. MSI status plays an important role in risk stratification and personalized treatment for GC patients [10]. In addition, MSI is associated with the genetic mechanisms driving Lynch syndrome (LS) carcinogenesis, the most common genetic disease leading to CRC [11]. However, MSI cannot fully identify all patients who may benefit from ICI treatment. Another biomarker, tumor mutational burden (TMB), complements with MSI in predicting ICI efficacy. TMB refers to the number of mutations detected per million nucleotides in tumor cells and is a pan cancer genomic biomarker associated with the efficacy of checkpoint inhibitors [12, 13]. Among various types of cancer, higher TMB is associated with prolonged overall survival of patients after immunotherapy. Therefore, CRC and GC patients associated with high TMB may have a higher chance of receiving immunotherapy [14]. However, traditional biomarkers prediction methods are costly, time-consuming, and complex to operate. Developing efficient biomarkers prediction methods are crucial for neoadjuvant therapy.

At present, deep learning methods have been widely applied in various of oncology, including tumor detection [15–19], tumor histological classification [20–22], and biomarker or prognostic prediction [17, 23–27]. For instance, Schirris *et al.* [28] proposed DeepSMILE, a weakly supervised learning method based on deep learning, which was applied to homologous recombination deficiency (HRD) and MSI prediction tasks in hematoxylin and eosin (H&E)-stained images. Similarly, Sadhwani *et al.* [29] developed a deep learning system that can both classify histological patterns of lung adenomas and predict TMB status using Whole-Slide Images (WSIs) with de-labeled H&E-stained images. However, the complexity of cancer causes [30] and technical limitations make it challenging to achieve a comprehensive diagnosis based on single feature information. In the past, many studies have often combined H&E-stained image features with multi-omics data features or clinical features to build predictive models, and their research has achieved certain results [31, 32]. For example, Qiu *et al.* [33] developed a new deep learning framework for predicting MSI status using only H&E-stained images and demonstrated that integrating H&E-stained images with molecular data improved prediction accuracy compared to using H&E-stained images alone. Huang *et al.* [34] proposed a multimodal deep learning model based on residual networks to predict TMB status directly from histopathological images and clinical data. Furthermore, the TME has been shown to play a key role in cancer development and progression, making it a promising target for cancer treatment strategies [35, 36]. Analyzing the composition of the tumor ecosystem and the relationship between different cell types is of great significance for understanding cancer biology and optimizing treatment approaches [37]. Notably, conventional image features alone inadequately resolve spatial heterogeneity within the TME, necessitating integration of histopathological patterns with nuclear morphometric data for accurate MSI/TMB prediction in gastrointestinal cancers.

Based on the feasibility of H&E-stained images to predict tumor markers and the importance of TME for immunotherapy, this study proposes a new deep learning predictive model framework. The framework integrates nuclear segmentation features (via Hover-Net) with image features (via CLAM) through Multimodal Compact Bilinear Pooling (MCB) fusion, capturing both tissue architecture and cellular-level information. This dual-branch design addresses the limitation of single-modality models in characterizing tumor heterogeneity and improves the performance of six deep learning prediction models in accurately predicting the MSI and TMB states of CRC and GC. However, this approach increases computational complexity due to parallel feature extraction pipelines. We mitigate this by: (i) Optimizing GPU memory usage via patch-wise processing; (ii) Employing MCB for dimensionality reduction rather than concatenation. In addition, we analyze the differences in cell composition between different cancer types and clinical groups, evaluate the effect of adding nuclear segmentation features to improve prediction performance, reveal the important role of cells in the TME, and deepen our understanding of TME. By addressing the shortcomings of traditional models in insufficient characterization of the microenvironment, it provides a new tool for immunotherapy screening.

## Materials and Methods

### Ethnical statement

The patient data of this research were retrieved following the approval of the Ethics Committee of the China-Japan Friendship Hospital (Approval No. 2024-KY-253).

### Data collection

#### TCGA image data

All H&E-stained whole-slide images from GC and CRC patients were obtained from The Cancer Genome Atlas (TCGA; https://portal.gdc.cancer.gov/). We retained only high-resolution (×40 magnification) images, excluding those with other magnifications. Images exhibiting poor quality—including uneven staining, blurring, or excessive bubbles—were excluded following manual assessment by a board-certified pathologist. We further excluded cases with missing MSI/TMB labels or incomplete clinical data. This yielded 350 GC and 376 CRC images for MSI prediction, and 400 GC and 387 CRC images for TMB prediction.

### Independent validation set

In this study, H&E staining images of 83 CRC patients in China-Japan Friendship Hospital were collected as an independent validation set, including 20 microsatellite instability-high (MSI-H) cases and 63 microsatellite stability (MSS) cases.

### Image data preprocessing

Due to the large number of pixels in WSI, preprocessing of the image data is necessary. First, the collected image data are divided into regions of interest (ROIs). These ROIs are then further segmented into 512 × 512 pixel image patches using OpenSlide. Patches with more than 30% blank areas are discarded. Subsequently, the selected patches undergo color normalization using the Macenko method to eliminate color variations caused by staining differences among different tissue sections. This step enhances the comparability and consistency of the images, ensuring that the model achieves better generalization performance across data from diverse sources.

### Image feature extraction

Extracting pathological image features efficiently and accurately is the key to build a high-performance prediction model. The CLAM model can effectively capture subtle differences in tissue regions through automatic segmentation and feature extraction of image regions, thereby providing high-quality feature representations for subsequent cancer analysis and prediction. Therefore, the CLAM model is employed in this study to extract image features. First, the entire H&E-stained image is automatically segmented to identify tissue regions. Next, the ResNet50 model pretrained on ImageNet is used to extract image features. Since the input data size for the ResNet50 network is 224 × 224 × 3, the patch size needs to be resized to the required dimensions before input. Subsequently, through the average pooling layer after the third residual block, each 512 × 512 patch is converted into a 1024-dimensional feature vector, with a batch size of 128 per GPU.

### Cell nucleus segmentation feature extraction

In cancer pathology research, nucleus segmentation and classification are critical steps for understanding the TME, assessing cellular heterogeneity, and predicting patient prognosis. The Hover-Net model is capable of simultaneously achieving precise nucleus segmentation and classification. It has been pretrained on the publicly available MoNuSAC dataset, which contains annotated nucleus data from multiple cancer types, enabling the model to generalize well to different types of pathological images. Therefore, the Hover-Net model is utilized to extract nuclear segmentation features. First, the preprocessed patch image data is used as input. The Preact-ResNet50 feature extractor is applied to extract high-level feature representations from the input images. Then, the extracted features are utilized for nucleus segmentation and classification, generating information such as nucleus locations and class probabilities. Additionally, an overlay map is created to visually display nucleus boundaries and classification results on the original image using color-coded overlays, where different colors represent different types of nuclei.

Using the segmentation and classification results generated by the Hover-Net model, for each processed patch, the cell contour features are calculated using skimage, yielding nuclear segmentation features that include cell type and morphological information. Subsequently, the cell types are converted into cell proportions, which involve statistically determining the proportion of each cell type within the WSI, thereby providing an intuitive representation of the cellular composition within the slide. Additionally, the average values of other nuclear segmentation features are computed to comprehensively describe the overall characteristics of cells in the WSI. This process facilitates the aggregation of patch-level nuclear segmentation features to the WSI level, offering a holistic perspective on cell composition and distribution across the entire slide.

### Feature fusion

In order to improve the performance of the model in predicting MSI and TMB, it is essential to fuse features containing different information. In this study, a MCB model is employed to combine the feature representation vectors derived from image features and cell nucleus segmentation features. This not only effectively avoids the curse of dimensionality, but also projects the original high-dimensional cross product results into a lower-dimensional space through nonlinear mapping. As a result, it significantly reduces computational complexity and resource consumption while retaining essential interaction information.

### Evaluation criteria

During the modeling process, in order to prevent overfitting, five-fold cross-validation was employed to determine the optimal parameters of the model and enhance its robustness. This study employed recall, specificity and Area Under the Receiver Operating Characteristic (ROC) Curve (AUC) to evaluate the model’s performance. The recall calculation formula is as follows (where TP, FP, TN, and FN represent true positive, false positive, true negative and false negative, respectively):


$$ Recall=\frac{TP}{TP+ FN} $$

$$ \mathrm{Specificity}=\frac{TN}{TN+ FP} $$

To address the limitation of missing per-fold validation results, we performed Bootstrap resampling (1000 iterations) on the mean performance metrics from five-fold cross-validation. Statistical significance was assessed via 95% confidence intervals and one-sided permutation tests (*P* < .05 considered significant).

### Computational optimization strategies

To mitigate the complexity of dual-branch feature extraction, the Hover-Net inference was distributed across four GPUs, the batch size was reduced to eight during training, and image features was extracted while nuclear segmentation runs.

## Results

### Deep learning framework for tumor marker prediction based on fusion cell nuclear segmentation features

**Figure 1:**
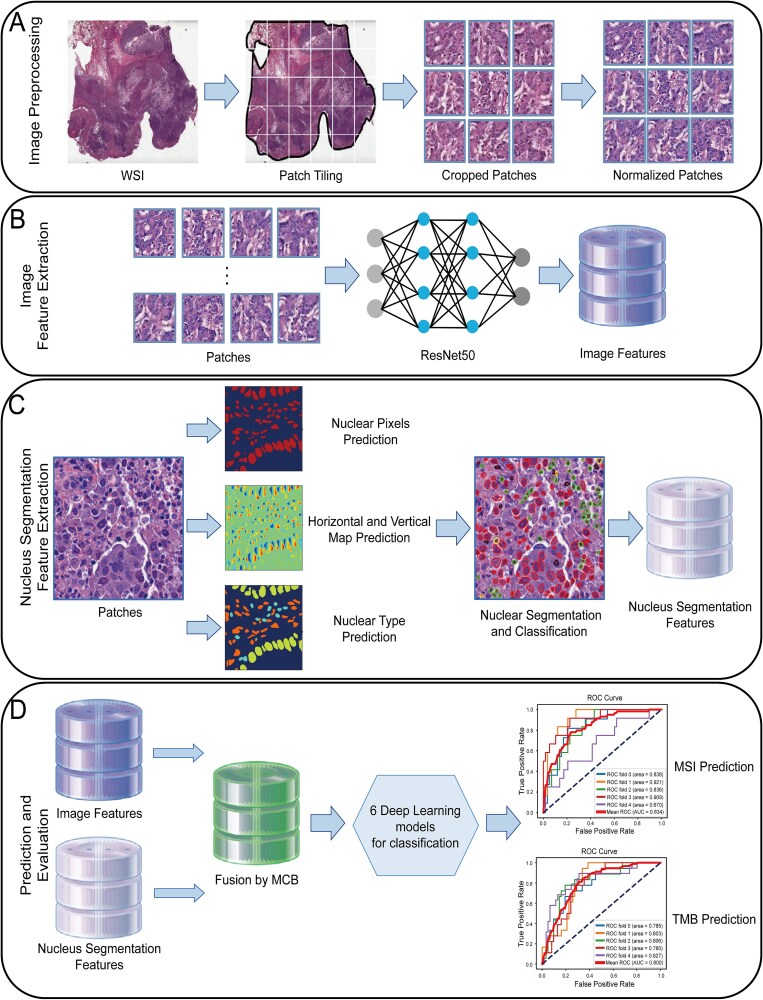
The study flow chart. A. Preprocessing of H&E-stained images: ROI division, image segmentation, color normalization. B. The ResNet50 residual network is used to extract image features. C. The Hover-Net model is used to extract nuclear segmentation features. D. The MCB model is used to fuse image features and nuclear segmentation features, and then six deep learning models are used to predict MSI and TMB states. The model was evaluated by five-fold cross-validation.

This study aimed to propose a deep learning model for automatically predicting MSI and TMB status in CRC and GC patients by analyzing H&E-stained images. The specific analysis process is shown in Fig. 1. First, H&E-stained image data for CRC and GC patients were obtained from the TCGA database and undergo preprocessing steps, including data cleaning and color normalization to ensure high data quality (Fig. 1A). Following preprocessing, The CLAM [38] model was utilized to automatically segment the H&E-stained images, identifying tissue ROIs. Subsequently, ResNet50 was applied to extract image features (Fig. 1B). Concurrently, the Hover-Net [39] model was employed to extract nuclear segmentation features (Fig. 1C), which further enriched the feature set. Next, the patient’s image features and nuclear segmentation features were combined using the MCB [40] model, and the resulting fused features were utilized to train the MSI and TMB prediction models (Fig. 1D). For each cancer type, five-fold cross-validation was applied during model training by fixing the random seed for the split of training and validation sets, while undersampling and stratified sampling techniques were employed to ensure the balance of the dataset and the consistent proportion of each category in every validation set with the overall dataset.

### The fusion of image features and cell nucleus segmentation features enhances the performance of MSI and TMB prediction models

**Figure 2:**
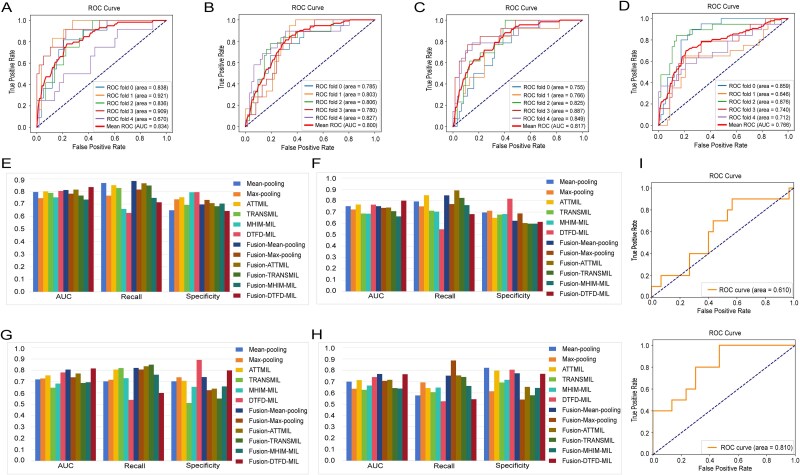
Performance comparison of different models. A. ROC curve for predicting MSI status of GC. B. ROC curve for predicting TMB status of GC. C. ROC curve for predicting MSI status of CRC. D. ROC curve for predicting TMB status of CRC. E. Histograms of performance evaluation indicators of different models when predicting MSI status of GC. F. Histograms of performance evaluation indicators of different models when predicting TMB status of GC. G. Histograms of performance evaluation indicators of different models when predicting MSI status of CRC. H. Histograms of performance evaluation indicators of different models when predicting TMB status of CRC. I. Results of independent validation: ROC curves for predicting MSI status of CRC based only on image features and based on fusion image features and nuclear segmentation features.

We separately predicted MSI and TMB status in CRC and GC patients. Six established multi-instance learning (MIL) architectures (Max-pooling, Mean-pooling, AttMIL [41], TransMIL [42], DTFD-MIL [43], and MHIM-MIL [44]) were selected as standard baselines for image-based biomarker prediction. To validate the utility of nuclear segmentation feature fusion, we conducted an ablation study (Fig. 2) comparing model performance with/without integrated nuclear features. This analysis revealed how architectural differences affect predictive accuracy when incorporating nuclear morphometrics, providing foundational insights for developing robust tumor-marker prediction systems. Crucially, to ensure a fair comparison, we only performed feature fusion without altering any other parameters of the models.

Based on the MSI prediction results for GC presented in Table 1 and Fig. 2E, the Fusion-DTFD-MIL model proposed in this study demonstrated the best performance, achieving an AUC of 0.8349 (Fig. 2A) and a recall of 0.7136. Compared to the original DTFD-MIL model, the AUC improved by 3%, the recall increased by 8%, and specificity decreased by 15%. In addition, the proposed Fusion-Mean-pooling, Fusion-Max-pooling, and Fusion-ATTMIL model have improved AUC and recall. The recall of the Fusion-TRANSMIL and Fusion-MHIM-MIL model improved by 2% and 9%, respectively, while the specificity decreased.

**Table 1:** MSI prediction results of GC.

Method	AUC	Recall	Specificity
Mean-pooling	0.7959	0.8667	0.6500
Max-pooling	0.7462	0.7667	0.7373
ATTMIL	0.8009	0.8500	0.7540
TRANSMIL	0.7883	0.8273	0.6930
MHIM-MIL	0.7529	0.6606	0.7936
DTFD-MIL	0.8038	0.6288	0.7943
Fusion-Mean-pooling	0.8114	0.8833	0.6964
Fusion-Max-pooling	0.7821	0.8152	0.7327
Fusion-ATTMIL	0.8146	0.8652	0.7073
Fusion-TRANSMIL	0.7676	0.8470	0.6821
Fusion-MHIM-MIL	0.7356	0.7485	0.7032
Fusion-DTFD-MIL	0.8349	0.7136	0.6447

According to the TMB prediction results for GC presented in Table 2 and Fig. 2F, the proposed Fusion-DTFD-MIL, Fusion-Max-pooling, and Fusion-TRANSMIL models achieved AUC values of 0.8003 (Fig. 2B), 0.7364, and 0.7074, respectively, with recall rates of 0.6813, 0.7702, and 0.8246. Compared to the original models, the AUC improved by 4%, 1%, and 2%, recall was increased by 13%, 2% and 11%, and specificity decreased by 20%, 7%, and 8%, respectively. In addition, the recall of Fusion-means-pooling, Fusion-ATTMIL, and Fusion-MHIM-MIL model were improved, while the specificity was decreased.

**Table 2:** TMB prediction results of GC.

Method	AUC	Recall	Specificity
Mean-pooling	0.7513	0.7930	0.6963
Max-pooling	0.7224	0.7497	0.7111
ATTMIL	0.7661	0.8480	0.6488
TRANSMIL	0.6868	0.7117	0.6772
MHIM-MIL	0.6850	0.7035	0.6826
DTFD-MIL	0.7649	0.5474	0.8172
Fusion-Mean-pooling	0.7513	0.8468	0.6228
Fusion-Max-pooling	0.7364	0.7702	0.6873
Fusion-ATTMIL	0.7394	0.8906	0.6029
Fusion-TRANSMIL	0.7074	0.8246	0.5971
Fusion-MHIM-MIL	0.6610	0.7608	0.5971
Fusion-DTFD-MIL	0.8003	0.6813	0.6132

Based on the MSI prediction results for CRC in Table 3 and Fig. 2G, the proposed Fusion-DTFD-MIL model demonstrated the best performance, achieving an AUC of 0.8162 (Fig. 2C). This represented a 4% improvement over the original DTFD-MIL model, significantly outperforming several methods that relied solely on image features, showing its good predictive capability and recall has also been improved. Additionally, the Fusion-Max-pooling, Fusion-Mean-pooling, Fusion-ATTMIL, Fusion-TRANSMIL, and Fusion-MHIM-MIL models all showed varying degrees of improvement compared to their original models, with average increases of 3% in AUC and 6% in recall.

**Table 3:** MSI prediction results of CRC.

Method	AUC	recall	Specificity
Mean-pooling	0.7194	0.7022	0.7025
Max-pooling	0.7274	0.7165	0.7380
ATTMIL	0.7550	0.8066	0.7071
TRANSMIL	0.6459	0.8198	0.5108
MHIM-MIL	0.6825	0.7297	0.6534
DTFD-MIL	0.7812	0.5385	0.8920
Fusion-Mean-pooling	0.8061	0.8209	0.7402
Fusion-Max-pooling	0.7380	0.8066	0.6239
Fusion-ATTMIL	0.7718	0.8352	0.6372
Fusion-TRANSMIL	0.6878	0.8495	0.5502
Fusion-MHIM-MIL	0.6945	0.7604	0.6574
Fusion-DTFD-MIL	0.8162	0.6000	0.7988

For TMB predictions in CRC, as shown in Table 4 and Fig. 2H, the proposed Fusion-Max-pooling, Fusion-Mean-pooling, Fusion-ATTMIL, Fusion-TRANSMIL, and Fusion-DTFD-MIL models exhibited improvements in both AUC and recall compared to the original models using only image features, while specificity decreased. The Fusion-Mean-pooling model achieved the highest AUC of 0.7666 (Fig. 2D), a 7% increase, and a recall of 0.75521, a 17% improvement. Although the AUC of the Fusion-MHIM-MIL model did not increase, its recall improved by 2% compared to the MHIM-MIL model.

**Table 4:** TMB prediction results of CRC.

Method	AUC	recall	Specificity
Mean-pooling	0.6992	0.5774	0.8213
Max-pooling	0.6360	0.6921	0.6138
ATTMIL	0.7125	0.6421	0.7969
TRANSMIL	0.6265	0.6063	0.6913
MHIM-MIL	0.6645	0.6468	0.7155
DTFD-MIL	0.7394	0.5258	0.8048
Fusion-Mean-pooling	0.7666	0.7521	0.7727
Fusion-Max-pooling	0.7061	0.8863	0.5408
Fusion-ATTMIL	0.7152	0.7542	0.6530
Fusion-TRANSMIL	0.6421	0.7405	0.5790
Fusion-MHIM-MIL	0.6378	0.6611	0.6439
Fusion-DTFD-MIL	0.7645	0.5442	0.7682

These results indicated that models based on the fusion of image features and nuclear segmentation features significantly outperform deep learning methods relying solely on image features.

In addition, all improvements in AUC for fusion models were statistically significant (*P* < .05 by Bootstrap resampling), which proved that the fusion strategy was significantly better than the single feature method at the statistical level, and provided a more reliable quantitative basis for clinical prognosis prediction.

### External independent validation

We applied identical preprocessing procedures, including patch segmentation and color normalization, to an independent validation cohort comprising 83 CRC patients from China-Japan Friendship Hospital. This cohort consisted of 20 MSI-H and 63 MSS cases. The DTFD-MIL model, which had exhibited superior predictive performance during training, was selected for external validation. On this independent set, the model utilizing only image features achieved an AUC of 0.61, with a recall of 0.70 and a specificity of 0.57. In contrast, the model integrating both image features and nuclear segmentation features attained an AUC of 0.81, a recall of 0.80, and a specificity of 0.70, corresponding to improvements of 20%, 10%, and 13%, respectively. The corresponding ROC curves are presented in Fig. 2I. These results underscore the value of incorporating nuclear segmentation features, which not only enhance predictive accuracy but also bolster model generalizability. The consistent performance observed across both cross-validation and independent external testing suggests robust generalization capability across heterogeneous data sources, including variations in population ethnicity, scanning equipment, and institutional protocols.

### Differential analysis of cell composition across cancer types

**Figure 3:**
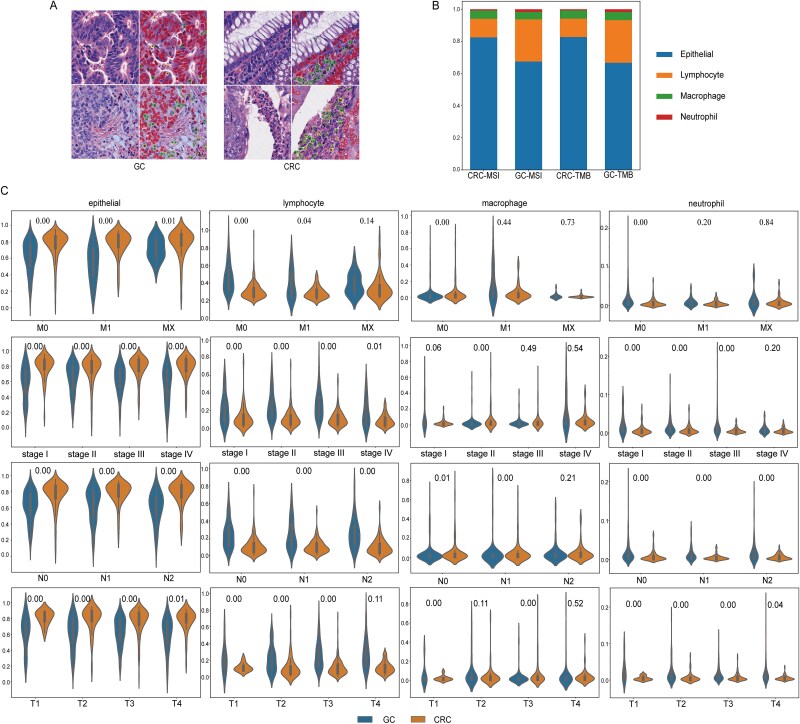
The differences in cell composition between different cancers. A. Cell nucleus segmentation map. Green dot represents lymphocytes, red dot represents epithelial cells, black dot represents macrophages, and yellow dot represents neutrophils. The left image shows GC, and the right image shows CRC. B. Columnal stacking plots of cell type proportions. C. Violin plots of differences in cell type proportions in different cancers.

This study conducted a differential analysis of cell composition. The nuclear segmentation results for CRC and GC are shown in Fig. 3A.

When comparing the proportions of cell types between the two cancers, we observed that while the cell composition was similar, the proportions of specific cell types differed significantly, as illustrated in Fig. 3B. Specifically, epithelial cells were dominant in both GC and CRC, accounting for ~67% in GC and 83% in CRC. Lymphocytes constituted ~26% of GC and 12% of CRC, and their proportion was significantly higher in GC than in CRC. This finding aligned with established biological mechanisms within the TME. Macrophages and neutrophils were present in relatively low proportions in both cancers (~5% and 1%, respectively).

To further quantify the differences in cell proportions between the two cancer types, we conducted the Mann–Whitney U test. *P* < .05 were considered statistically significant. The results revealed significant differences in the distribution of cell types between CRC and GC, as shown in Fig. 3C, underscoring the biological distinctions between these cancers.

In both GC and CRC, epithelial cells exhibited significant differences across the clinical feature pathological staging M (M0, M1, and MX). Lymphocytes showed significant differences between M0 and M1, while macrophages and neutrophils demonstrated significant differences in M0. Epithelial cells and lymphocytes exhibited significant differences across the clinical feature tumor staging stage (stage I to stage IV). Macrophages showed significant differences in stage II, and neutrophils exhibited significant differences in stage I, stage II, and stage III. Epithelial cells, lymphocytes, and neutrophils displayed significant differences across the clinical feature pathological staging N (N0, N1, and N2), while macrophages showed significant differences in N0 and N1. Epithelial cells and neutrophils exhibited significant differences across the clinical feature pathological staging T (T1, T2, T3, and T4), lymphocytes showed significant differences in T1, T2, and T3, and macrophages demonstrated significant differences in T1 and T3.

### Differential analysis of cell composition between different clinical groups

This study conducted a differential analysis of cell composition between different clinical groups in order to deeply understand the dynamic changes in the TME.

**Figure 4:**
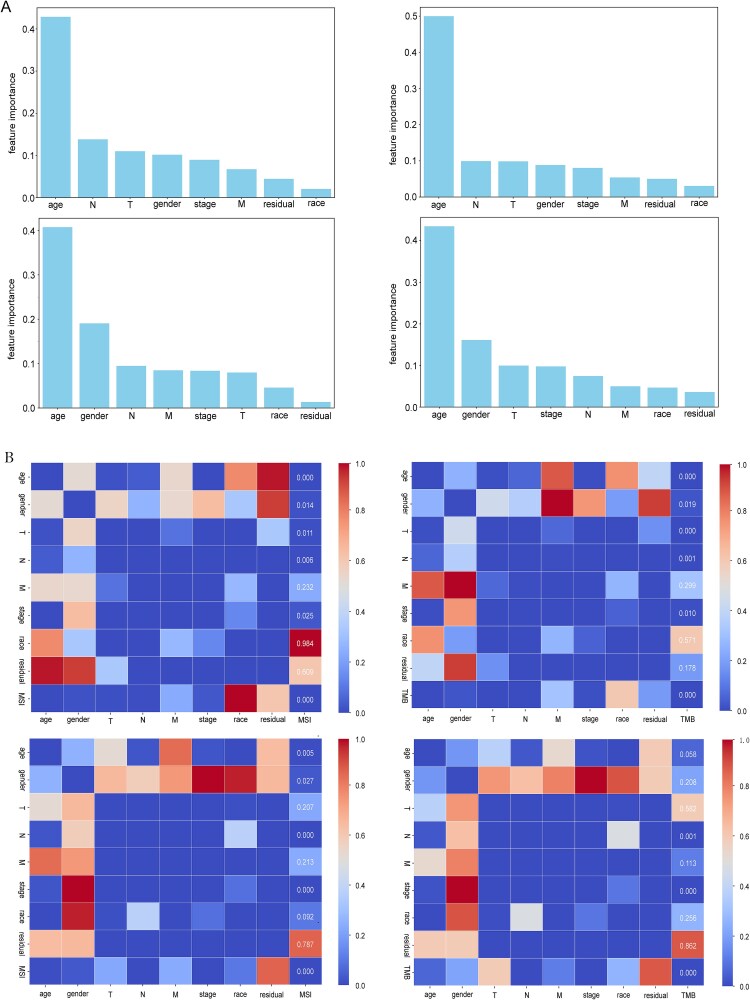
Clinical feature importance ranking and correlation testing. A. Feature importance ranking chart. From left to right, the feature importance ranking diagram for predicting MSI for CRC, TMB for CRC, MSI for GC, and TMB for GC is shown. B. Correlation test chart. From left to right, the correlation test charts for predicting MSI for CRC, TMB for CRC, MSI for GC, and TMB for GC are shown.

First, clinical data from CRC and GC patients were obtained from TCGA and matched with H&E-stained image data. Missing values in clinical data were imputed using the random forest method. The importance of preprocessed clinical features was then ranked using a decision tree approach, quantifying the contribution of each feature to the model’s predictive performance. The Gini index histogram, sorted by feature importance, is shown in Fig. 4A. The top six clinical features related to MSI and TMB in CRC and GC were identified as age, gender, T, N, M and stage. A Spearman correlation test was performed to visualize the strength of the relationship between clinical features and TMB/MSI status, with the correlation heatmap shown in Fig. 4B. Features with *P* < .05 were considered statistically significant, further confirming the close relationship between different clinical features and TMB and MSI states. Based on these results, six clinical features were selected for further analysis, including age, gender, T, N, M and stage.

**Figure 5:**
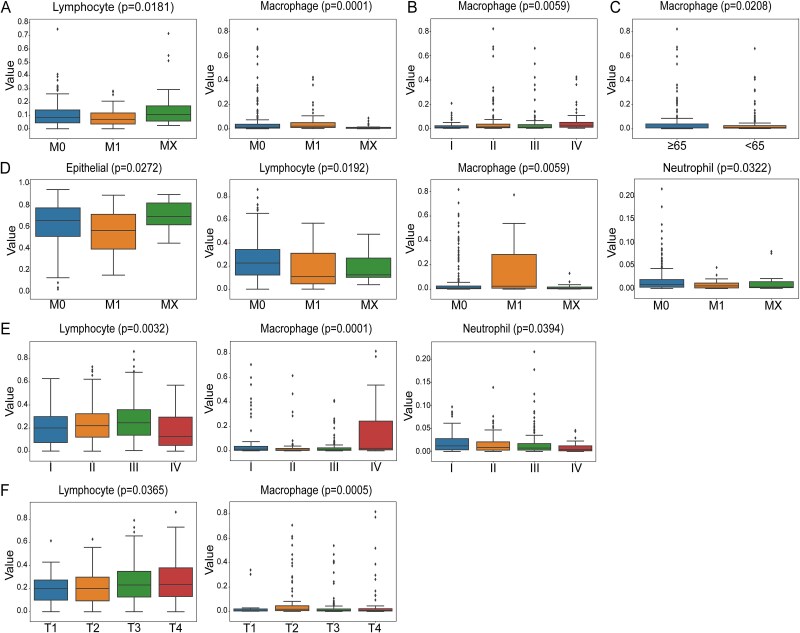
Differences in cellular composition between different clinical groups. A. Boxplot of differences in cell composition of CRC in clinical group M. B. Boxplot of differences in cell composition of CRC in clinical group stage. C. Boxplot of differences in cell composition of CRC in age clinical groups. D. Boxplot of differences in cell composition of GC in clinical group M. E. Boxplot of differences in cell composition of GC in clinical group stage. F. Boxplot of differences in cell composition of GC in clinical group T.

Next, we used the Kruskal–Wallis test to analyze differences in cell composition between different clinical groups. *P* < .05 were considered statistically significant. Significant variations in cell composition were observed among different clinical groups, as illustrated in Fig. 5. In CRC, significant differences were observed in lymphocytes and macrophages within the clinical group of pathological staging M (Fig. 5A), while macrophages showed significant differences in the clinical group of tumor staging stage (Fig. 5B). Additionally, macrophage cells exhibited significant differences in the clinical group of age (Fig. 5C). In GC, significant differences were observed in epithelial cells, lymphocytes, macrophages, and neutrophils within the clinical group of pathological staging M (Fig. 5D). lymphocytes, macrophages, and neutrophils exhibited significant differences in the clinical group of tumor staging stage (Fig. 5E), while Lymphocytes and macrophages showed significant differences in the clinical group of pathological staging T (Fig. 5F).

## Discussion

Deep learning approaches have demonstrated significant utility across diverse oncology applications, including protein prediction, drug response modeling, and therapeutic target identification [45–48]. Addressing the critical need for cost-effective biomarker testing and overcoming single-modality limitations, we developed a nuclear segmentation feature fusion model to enhance prediction accuracy and interpretability of MSI/TMB status in GC and CRC. Our framework systematically compared three feature paradigms: (i) histopathology image features alone, (ii) fused image features, and (iii) nuclear segmentation features with image fusion. Results demonstrated that integrating nuclear segmentation features substantially improved MSI/TMB prediction performance in both cancer types. Despite limited external validation cohort size (*n* = 83) and variations in population ethnicity, the model maintained robust discriminative capacity (AUC: 0.81). Furthermore, cellular composition analysis revealed significant variations across cancer types and clinical subgroups, with strong correlations between immune cell spatial distributions and clinical features. These findings not only reflect fundamental biological heterogeneity between GC and CRC, but also elucidate dynamic tumor-immune interactions within the TME. Our work thereby provides histopathological rationale for differential immunotherapy responses and supports cellular spatial signatures as promising predictive biomarkers [49, 50].

In summary, our nuclear segmentation feature fusion model provides a more comprehensive representation of tumor histopathology, significantly enhancing MSI/TMB prediction performance while demonstrating the critical value of incorporating nuclear morphological data. The framework revealed fundamental biological differences in the TME between GC and CRC, particularly in immune-stromal architecture. Crucially, this approach demonstrates broad clinical applicability by simultaneously predicting multiple biomarkers for two high-incidence gastrointestinal malignancies using cost-effective, widely available H&E slides. These advantages position our method as a scalable tool for advancing precision oncology in digestive tract cancers.

This study has several limitations. First, nuclear segmentation using Hover-Net increased computational overhead compared to image-only models. Future work will pursue integration of lightweight segmentation architectures and explainable AI techniques to mitigate these constraints. Second, cohort size was limited by data availability; the external validation set comprised only 83 CRC samples, potentially affecting performance estimation precision. Model generalizability to rare histological subtypes may be constrained by their underrepresentation in training data. We plan to expand cohorts through multicenter collaborations and conduct prospective clinical trials to validate clinical utility. Third, while our fusion of image and nuclear features provided predictive value, it does not fully capture molecular-level tumor alterations or nuanced tumor-microenvironment interactions. Future research will integrate multi-omics data to elucidate relationships between cellular phenotypes, functional states, and their mechanistic roles within the tumor ecosystem.

## Conclusion

In this study, we provide a accurate, cost-effective, and scalable tool for MSI/TMB screening, leveraging universally available H&E slides to potentially reduce the need for more expensive and time-consuming genomic tests. By successfully translating nuclear morphological patterns into predictive biomarkers, this work paves the way for more accessible precision oncology in digestive tract cancers. Future efforts will focus on integrating multi-omics data, adopting more efficient segmentation models to reduce computational overhead, and validating the approach in larger, prospective multi-center trials to further solidify its clinical utility.

**Key Points**

- We developed a dual-branch deep learning framework that integrates tissue-level histopathological features from H&E images with morphometric nuclear features from segmented nuclei, substantially improving MSI/TMB prediction accuracy in gastric (GC) and colorectal cancer (CRC).
- A computationally efficient pipeline for simultaneous whole-slide image (WSI) feature extraction and nuclear segmentation was established, enabling interpretable, H&E-stain-based biomarker prediction without requiring additional assays.
- Quantitative analysis revealed compartment-specific cellular composition differences between GC and CRC microenvironments, and across clinical stages, providing mechanistic insights into tumor progression dynamics.
- Model generalizability was confirmed using a prospective external cohort from China-Japan Friendship Hospital, demonstrating consistent real-world performance.

## Data Availability

The code supporting the findings of this study is publicly available at: https://github.com/hjy212/DTFD-Cell-MIL. The datasets analyzed in this article are accessible from The Cancer Genome Atlas (TCGA) via: https://portal.gdc.cancer.gov/. Additionally, data from the China-Japan Friendship Hospital are available upon reasonable request.
